# Intercellular communication in the cumulus–oocyte complex during folliculogenesis: A review

**DOI:** 10.3389/fcell.2023.1087612

**Published:** 2023-01-19

**Authors:** Jun Xie, Xiao Xu, Suying Liu

**Affiliations:** ^1^ Reproductive Medicine Center, Zhongshan Hospital, Fudan University, Shanghai, China; ^2^ Department of Obstetrics and Gynecology, Zhongshan Hospital, Fudan University, Shanghai, China

**Keywords:** cumulus-oocyte complex, intercellular communication, folliculogenesis, transzonal projections, gap junctions, microvilli, extracellular vesicles, cell membrane fusion

## Abstract

During folliculogenesis, the oocyte and surrounding cumulus cells form an ensemble called the cumulus-oocyte complex (COC). Due to their interdependence, research on the COC has been a hot issue in the past few decades. A growing body of literature has revealed that intercellular communication is critical in determining oocyte quality and ovulation. This review provides an update on the current knowledge of COC intercellular communication, morphology, and functions. Transzonal projections (TZPs) and gap junctions are the most described structures of the COC. They provide basic metabolic and nutrient support, and abundant molecules for signaling pathways and regulations. Oocyte-secreted factors (OSFs) such as growth differentiation factor 9 and bone morphogenetic protein 15 have been linked with follicular homeostasis, suggesting that the communications are bidirectional. Using advanced techniques, new evidence has highlighted the existence of other structures that participate in intercellular communication. Extracellular vesicles can carry transcripts and signaling molecules. Microvilli on the oocyte can induce the formation of TZPs and secrete OSFs. Cell membrane fusion between the oocyte and cumulus cells can lead to sharing of cytoplasm, in a way making the COC a true whole. These findings give us new insights into related reproductive diseases like polycystic ovary syndrome and primary ovarian insufficiency and how to improve the outcomes of assisted reproduction.

## 1 Introduction

The human body is a sophisticated assembly, in which the most basic unit of work is the cell. Interactions between cells enable the whole body including the reproductive system to function. The ovary is an important part of the female reproductive system and endocrine axis. In the mammalian ovary, the oocyte and adjacent somatic cells are interdependent and participate in follicular development and ovulation.

During embryonic development, primordial germ cells specialize and migrate to the germinal crest and form the ovary together with the mesodermal cells of the germinal crest, which further proliferate to form germ cell nests and eventually become primordial follicles (KlocJacek et al., 2016). Each primordial follicle contains an oocyte and a layer of ovarian granulosa cells surrounding the oocyte, and as the follicle grows and develops, its structure changes dynamically: the oocyte gradually increases in size, while the surrounding granulosa cells grow exponentially from the initial monolayer flattened pre-granulosa cells to stratified columnar cells (Xu et al., 2022). At the same time, they spatially differentiate into cumulus cells (CCs) and mural granulosa cells (MGCs) (Scott et al., 2018). At the secondary follicle level, the follicle is surrounded by a layer of theca cells. Follicle-stimulating hormone (FSH) and luteinizing hormone (LH) play important roles in the process of antral follicles to ovulation (Edson et al., 2009). FSH promotes the growth, hormone synthesis, and receptor expression of granulosa cells. The increasing LH receptor (LHR) also responds to the LH peak before ovulation and activates the final ovulation. Since the primary follicle, the oocyte begins to secret a family of glycoproteins that assemble into an extracellular coat, termed the zona pellucida (ZP), which physically separates the oocyte from the bodies of the adjacent cumulus cells.

Throughout this process, the cumulus cells remain close to the oocyte and together they are called the cumulus-oocyte complexes (COC). Numerous communications, which occur in the COC, maintain the growth of the oocyte and follicular hemostasis (Russell et al., 2016). The integrity of the ZP is critical for intercellular communications that mostly take place there (Gupta, 2018; Wang et al., 2019). Advanced observation techniques have enabled more connection structures to be discovered. This critical review of the literature focuses on recent progress and characterizes the morphology and function of COC. Advances in this field can possibly help us better understand mechanisms of folliculogenesis and pathogenesis of reproductive diseases, as well as guide future approaches for improving assisted reproduction techniques.

## 2 Morphology of intercellular communication in COC

### 2.1 Transzonal projections (TZPs)

CCs have highly specific trans-cytoplasmic processes, for example, they have thin cytoplasmic TZPs that protrude through the ZP to the oocyte (Albertini et al., 2001, Albertini, Combelles, Benecchi, Carabatsos). Unlike traditional filopodia, in addition to cell-to-cell signaling, CCs provide the material basis for the growth and development of oocytes through TZPs (Clarke, 2018). This special structure discovered by scientists over 50 years ago (Hadek, 1965) has become a research hotspot. Because of the development of observation technology, the secret of this mini channel has been gradually revealed. As specialized filopodia, TZPs contain an F-actin backbone and some contain a tubulin backbone (El-Hayek et al., 2018), but the ratio and function of the two are not yet fully understood and further studies are necessary.

A recent 3D-reconstruction study in mice by Baena and Terasaki (Baena and Terasaki, 2019) used new methods for serial section electron microscopy and showed that most TZPs branched and made gap junctions with each other rather than directly reaching the oocyte. They found that on average, every CC had about 30 TZPs that did not reach the surface of the oocyte, and 9 TZPs that reached the oocyte after forming a junction with other CCs. The lengths of connected TZPs were evidently larger than the average thickness of the ZP for about 4 μm. This makes it possible for connected TZPs to extend along the surface of the oocyte or even loop back to the ZP. Also, side-to-side, or end-to-end gap junctions were observed between TZP contact sites, either from the same CC or two different CCs. These findings indicate the fundamental structure as well as the complexity of TZPs. However, the detailed mechanism requires further research. Crucially, most TZPs that reach the oocyte surface are contacted by several oocyte microvilli.

Filopodia were first formed at the early primary follicle stage in the mouse, and a protein named MYO10 might participate in the morphogenesis of TZPs (Granados-Aparici et al., 2022). MYO10 could promote and maintain TZP formation. What’s more, the foci of MYO10 near cell membrane of CCs may represent the initiation sites of TZPs. It’s interesting that another study (Crozet et al., 2021) on mouse oocyte found MYO10 (termed as myosin-X in this paper) to play a role in the migration of the meiosis I spindle through F-actin, the same one in TZPs. More research is needed to elucidate the functions of this potentially critical protein in COC.

### 2.2 Types of junctions

Gap junctions (GJs) are one of the most studied forms of intercellular communications in the mammalian ovary (Gershon et al., 2008; Loiselle et al., 2013; Winterhager and Kidder, 2015). GJs are found on the tips of TZPs that contact the oocyte plasma membrane. They are composed of multidomain transmembrane proteins, the main components of which are connexins (CXs). The two important connexins in COC are CX43, which is distributed in granulosa cells, and CX37, which is distributed in oocytes and CCs (Kidder and Mhawi, 2002; Beyer and Berthoud, 2018). It is widely thought that they participate in the communications of different cells, CX43 in CC-to-CC and CX37 in CC-to-oocyte communications (Simon et al., 2006; De los Reyes et al., 2020). This is based on the distribution and properties of the channels such as composition. Gerald M. Kidder et al. suggested that transgenic mice with GJA1 (CX43 encoding gene) controlled by the Zp3 gene promoter crossed with CX37-null mutant mice could restore the formation of COC, development, and maturation of oocytes, and fertility (Li et al., 2007). This suggests that it is physiologically possible for CX43 to perform the functions of CX37. However, this study was limited to mice, and although both connexins can transmit some basic molecules and ions, they are essentially different, for example, CX37 channels are more stringent than CX43 in transmitting larger molecules and certain ions (Beyer and Berthoud, 2018). More studies are needed to confirm whether CX43 substitution has the same effect physiologically as CX37 and if oocyte quality is affected.

Tight junctions (TJs) and adherens junctions (AJs) are also mentioned in the development of COC (Schuster et al., 2004; Mora et al., 2012). TJs consist of transmembrane adhesion molecules such as claudins, occludins, and junctional adhesion molecules (JAMs). Cytoplasmic scaffolding proteins such as zonula occludens (ZO) and cingulin bind TJs directly to the actin cytoskeleton. AJs are typically more foundational than TJs and are thought to be involved in signaling events in addition to cell adhesion. The important adhesion molecules in AJs are cadherins and nectins (Niessen, 2007). TJs were present at the sites of cellular membrane apposition (Anderson and Albertini, 1976; Gilula et al., 1978), whereas ZO-1/2 were abundant in both oocytes and CCs (Cao et al., 2021). Mora et al. (Mora et al., 2012) found AJs alongside GJs, but no evidence suggests that mature TJs might play a role in follicle function and development. We need further studies to clarify the presence and meaning of TJs and AJs in COC.

### 2.3 Microvilli

Microvilli are found on the surface of many cell types including oocytes (Runge et al., 2007). The increase of microvilli on the surface of oocytes was closely related to the appearance of ZP, and their shape changed after meiosis resumed (Yu et al., 2010; Benammar et al., 2017). At the germinal vesicle stage, microvilli are regular and parallel, homogeneously distributed on oocyte membrane. When it comes to metaphase II stage, they are larger, shorter, and more disorderly distributed.

As previously described (Baena and Terasaki, 2019), TZPs between oocytes and CCs are found to be closely associated with 3–6 oocyte microvilli, which form a “clumped phenomenon”. The contacts between TZPs and microvilli are not gap junctions. Another study used endogenous-fluorescent tracing mouse models to investigate the formation, structure, and role of oocyte microvilli (Zhang et al., 2021a). Microvilli are shaped like mushrooms, consisting of a slender handle and a swollen vesicle tip. They are formed gradually as follicles grow but do not increase in size after follicles are mature. The microvilli contents were derived from the endoplasmic reticulum. And the vesicle rupture was also dynamically captured at the head of microvilli. The oocyte could take advantage of this to positively communicate with somatic cells nearby, instead of just passively receiving molecules, and even successfully fuse with gametes (Inoue et al., 2020).

### 2.4 Extracellular vesicles (EVs)

During follicular development, in addition to the connections mentioned above, there is mounting evidence that granulosa cells also secrete membrane-enclosed vesicles that affect surrounding cells, called EVs. EVs are abundant in the human body and many macromolecules such as miRNAs, lncRNAs, and proteins are transported through them (Caballero et al., 2015; Abels and Breakefield, 2016; van Niel et al., 2018). It is commonly thought that the 3 main types of vesicles are: exosome, apoptotic body, and ectosome (or shedding microvesicle). They differ in size, membrane composition, and biosynthetic pathways. Microvesicles and exosomes were identified in follicular fluid, and miRNAs and proteins were found in them (da Silveira et al., 2012). The average diameter of total EVs isolated from human follicular fluid is about 200 nm, with microvesicles ranging from 100–1000 nm and exosomes ranging from 30–150 nm (Neyroud et al., 2022). Further studies confirmed that these miRNAs and proteins were also present in CCs, suggesting that these vesicles were synthesized and secreted by CCs. Co-culture of EVs extracted from the follicular fluid with CCs could increase their proliferative ability. Consequently, CCs can also act as recipients that ingest vesicles. Meanwhile, EVs were observed around the tips of TZPs, suggesting that some EVs might also be released by TZPs (Macaulay et al., 2014; Macaulay et al., 2016). The number of EVs gradually increased as the follicles developed, and their contents also changed (Marchais et al., 2022).

With Cryo-TEM, Neyroud AS et al. provided a basic morphological description of EVs (Neyroud et al., 2022). Interestingly, they found 10 subtypes of EVs with totally different structures, from normal single vesicles to double even triple vesicles. It’s likely that the internal cargos carried by EVs might shape the external forms. But because of the technical limitation, it’s rather hard to separate each type of EVs and confirm its content. Studies on follicular EVs so far were mainly completed through directly analyzing specific molecules from whole follicular fluid. Therefore, further studies are necessary to elucidate the morphology of EVs in follicular fluid with some imaging techniques like transmission electron microscopy.

As cell-secreted vesicles, their contents are the core mediator to make a difference. It’s commonly believed that there are three main types of extracellular vesicle cargos, including proteins, lipids and nucleic acids (Simpson et al., 2012; Kim et al., 2013). In the field of folliculogenesis, miRNAs are the mostly studied, with a variety of miRNAs and related pathways being identified (da Silveira et al., 2012; Santonocito et al., 2014; Diez-Fraile et al., 2014; Gebremedhn et al., 2020). Proteins are also well studied (Grzesiak et al., 2020; Uzbekova et al., 2020) with proteomic analysis. EVs can transport numerous ribosome and RNA-binding proteins. Recently, more studies focused on other molecules like lipids and lncRNAs (da Silveira et al., 2021; Bai et al., 2022). Obviously, EVs play an important role in the folliculogenesis.

### 2.5 Cell membrane fusion

**FIGURE 1:**
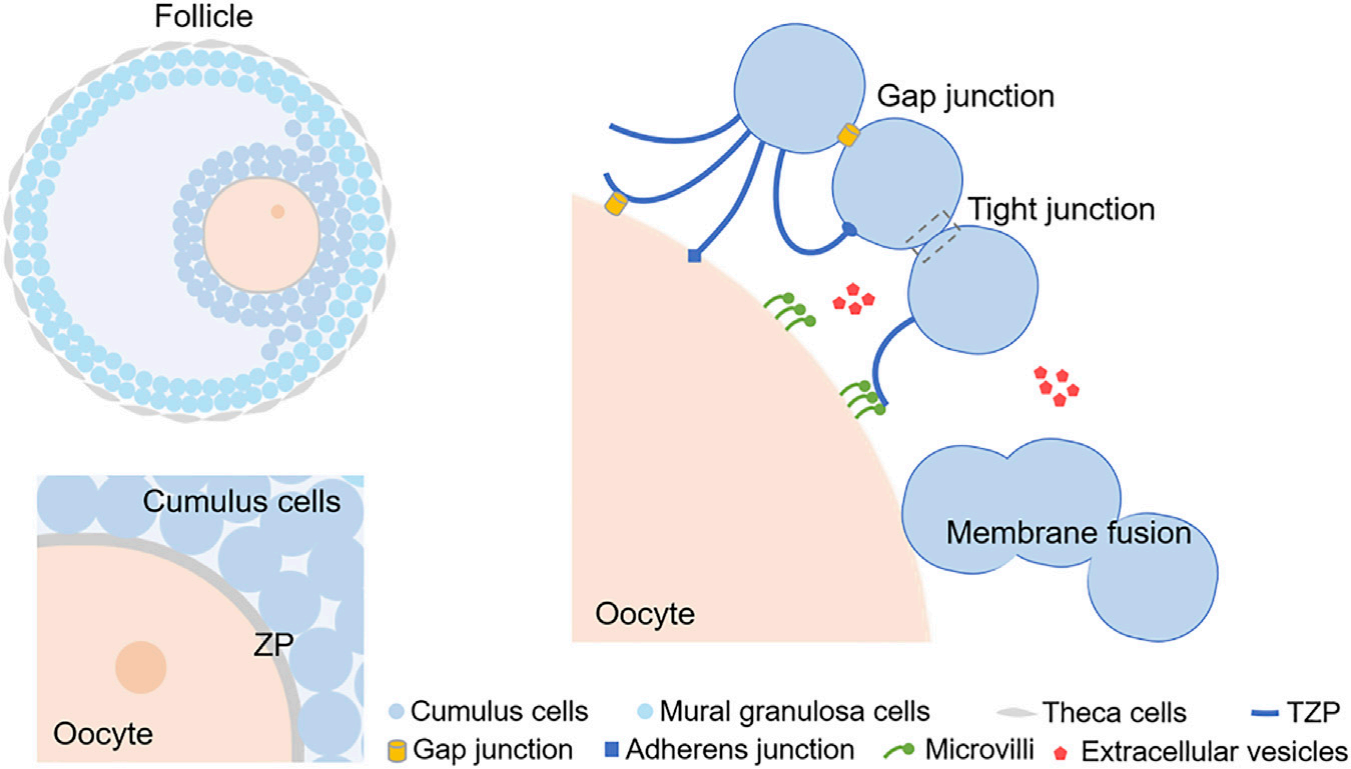
A schematic diagram exhibiting the intercellular communication in cumulus-oocyte complex. The simple diagram of cumulus oophorus in an antral follicle is showed at top left. The 2 layers of cells adjacent to the oocyte are CCs and the cells in the outer layers of the follicle are mural granulosa cells. ZP surrounds the plasma membrane of the oocyte, which is labeled at lower left. TZPs can protrude through the ZP to the oocyte and form connections. Microvilli appear on the surface of the oocyte, and they can either contact with TZPs or release EVs. EVs can also be released by CCs. Cell membrane fusion is another phenomenon occurring both in oocytes and CCs. CC, Cumulus cell; ZP, Zona pellucida; TZP, Transzonal projection; EV, extracellular vesicle.

**FIGURE 2:**
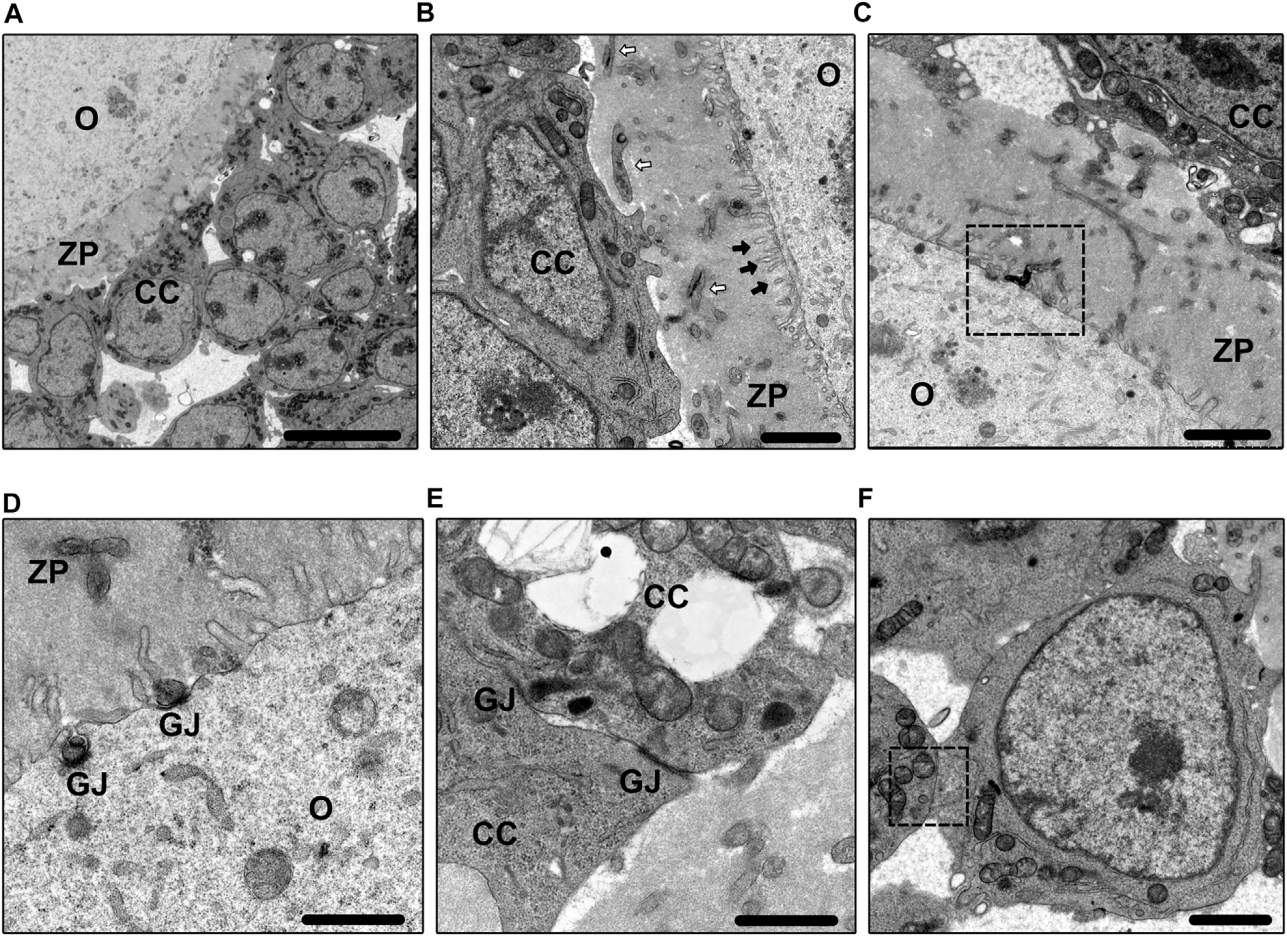
Electron microscope images. Figure 2A shows the basic morphology of COC in the primary follicle. Bar = 10 µm. Figure 2B shows the morphology of TZPs (white arrows) and microvilli (black arrows). Bar = 2 µm. Figure 2C shows the clumped phenomenon between TZPs and microvilli. Bar = 2 µm. Figure 2D shows the morphology of GJs between TZPs and oocyte. Bar = 1 µm. Figure 2E shows the morphology of GJs between CCs. Bar = 1 µm. Figure 2F shows the cell membrane fusion between CCs. Bar = 2 µm. O, oocyte; CC, cumulus cell; ZP, zona pellucida; TZP, transzonal projection; GJ, gap junction.

Fusion is involved in many cellular processes, and cell membrane fusion is a direct way of combining different cells to form an intact complex (Jahn et al., 2003; Martens and McMahon, 2008). The fusion of the oocyte and sperm has been well elucidated (Georgadaki et al., 2016), but the question of whether there is membrane fusion between oocytes and CCs remains. Recently, interaction and fusion between the membranes of oocytes and CCs have been reported (Komatsu and Masubuchi, 2018). Researchers used transgenic technology to construct a model expressing AcGFP1 (a monomeric green fluorescent protein) on the oocyte membrane only. AcGFP1 could not pass through the GJs because of its large molecular weight. Using fluorescence tracing, the positive projection of AcGFP1 was found to extend from the oocyte to the CCs, in contrast to TZPs (from CCs to oocyte). AcGFP1 was also detected in the membrane of CCs in both primary and secondary follicles. The expression of AcGFP1 was gradually increased with follicular development, but disappeared after ovulation. Under electron microscopy, AcGFP1 was transmitted by membrane fusion, rather than TZPs. It is likely that the COC share and use cytoplasm to deliver some macromolecules that cannot be transferred through GJs. Cell membrane fusion, together with microvilli, provide the fundamental structure for oocyte to positively communicate with CCs. The roles of cell membrane fusion in folliculogenesis require more research to confirm. In brief, the interactions in the follicles are certainly more complex than initially thought. In addition to TZPs, different junctions, microvilli, extracellular vesicles, and cell membrane fusion contribute to the fundamental structures of COC communication (Figure 1). Transmission electron microscope images of different types of intercellular communications are presented in Figure 2.

## 3 Functions of intercellular communication in COC

### 3.1 Effects on oocytes: Transfer of metabolites

The energy supply needed for oogenesis is derived from intercellular communication. It has been proven that CCs can transfer ATP directly to the oocyte through GJs and shutting down GJs could lead to a decrease of ATP in oocytes (Saito et al., 1994; Downs, 1995; Downs and Utecht, 1999; Su et al., 2009; Dalton et al., 2014). Glucose metabolism, a critical process, is accomplished mainly through glycolysis, pentose phosphate pathway (PPP), and polyol pathway. As a key enzyme in the glycolysis, the phosphofructokinase (PFK) in CCs has a higher activity than that in oocyte (Cetica et al., 2002; Roberts et al., 2002). Thus, there is a more rapid metabolism of glucose to pyruvate in CCs *via* PFK activity. These pyruvates can be transferred to the oocyte from CCs during maturation. (Harris et al., 2009; Sutton-McDowall et al., 2010). NADPH and ribose 5-phosphate are produced through the PPP. NADPH can be transferred into the oocyte to participate in biosynthesis and maintain cytoplasmic integrity and redox status. Ribose 5-phosphate can form phosphoribosyl pyrophosphate (PRPP), which is essential for *de novo* and salvage nucleotide synthesis in oocytes (Richani et al., 2021). Recently, Kang et al. (Kang et al., 2021) proposed that the PPP of three pathways was the most important to oogenesis in zebrafish.

Under normoglycemic conditions, the polyol pathway accounts for very little glucose metabolism in somatic cells mainly because of the low affinity of aldose reductase for glucose. While aldose reductase is expressed in rat ovarian granulosa cells and oocytes, and sorbitol dehydrogenase is highly expressed in oocytes (Kaneko et al., 2003). These two enzymes are indispensable for the conversion of glucose to fructose. Although CCs have high levels of glycolytic activity that supply oocytes with metabolites for oxidative phosphorylation, the expression of these enzymes suggests that they can also convert glucose to fructose, which provides oocytes with an alternative substrate for energy production. To date, the levels of sorbitol and fructose in COCs have not been measured. Zhang Y et al. (Zhang et al., 2021b) found that excess sorbitol accumulation during *in vitro* maturation (IVM) of aged mouse oocytes altered the intracellular redox balance, potentially decreasing oocyte quality. The findings may contribute to a better understanding of these three pathways of human oogenesis.

Conversely, oocytes can influence the energy metabolism of CCs through oocyte secreted factors (OSFs) secretion. Current studies have shown that OSF effects on energy metabolism vary among species (Sugiura et al., 2005; Sugiura et al., 2007; Caixeta et al., 2013). Oocytes can secrete OSFs to regulate the synergistic effect of energy metabolism between oocytes and CCs.

### 3.2 Effects on oocytes: Meiosis

GJs are directly involved in oocyte meiosis. Cyclic adenosine monophosphate (cAMP) and cyclic guanosine monophosphate (cGMP) can be transmitted as second messengers from the granulosa cells to the oocyte through GJs (Shuhaibar et al., 2015; Jaffe and Egbert, 2017). These two substances play a key role in inhibiting meiotic resumption. Oocyte cAMP can be produced *in situ* (*via* G protein-coupled receptor 3, G proteins, and adenylate cyclase), and transmitted from CCs (Bornslaeger and Schultz, 1985; Bornslaeger et al., 1986; Webb et al., 2002; Vaccari et al., 2008). High levels of cAMP in the oocyte maintain meiotic arrest. cAMP inhibit the activity of CDK1 *via* PKA, thereby suppressing the activation of maturation-promoting factors, thus leading to a meiotic arrest (Pirino et al., 2009). Meanwhile, cGMP synergistically increases the intracellular concentration of cAMP by inhibiting phosphodiesterase hydrolysis (Conti et al., 2012). Recent studies on the cGMP regulatory pathway NPPC/NPR2 have shown that NPPC/NPR2 and related signals could increase the intracellular cGMP level in CCs, thus maintaining meiotic arrest in oocytes (Celik et al., 2019; Ni et al., 2021).

Meiotic resumption also requires a lot of preparations including RNA transcription. A recent study proposed that EVs might also play an important role in transcription (Macaulay et al., 2014). Macaulay et al. (Macaulay et al., 2016) showed that RNAs (miRNA, lncRNA, etc.) synthesized by CCs are transported to TZPs and then to the oocyte *via* EVs. Their further study revealed that transcripts were aggregated in the polyribosomes of the oocyte, suggesting that they were further translated after entering the oocyte. These findings lead to the hypothesis that CCs contribute a portion of their RNAs to the oocyte to help it develop better; in this way, the oocyte can continue to grow even during transcriptional quiescence. Cutting off this RNA transmission can cause impaired oocyte development and a significant reduction in maturation rate, which would confirm our hypothesis. Another study revealed a membraneless compartment storing mRNAs around mitochondria in the germinal vesicle oocyte (Cheng et al., 2022). Combining these evidence, it’s likely that RNAs, including those transported from CCs, would aggregate in this compartment if the biosynthesis load is not high. And when the meiosis resumes they would be utilized conveniently.

Some scholars hypothesize that cells expel unnecessary metabolites through EVs, so studying the vesicles in the follicular fluid is somewhat misleading, but the debate about this continues. We think EVs are of special significance in the process of oogenesis, and more precise studies are needed to answer how and by what vesicles are excreted and absorbed, and with what effect. More effective trace imaging techniques are required to localize vesicles in the follicles.

### 3.3 Other effects on oocytes

Lipid metabolism also plays a key role in the homeostasis of the follicular microenvironment. During folliculogenesis, the follicular fluid contains a certain concentration of fatty acids, which can be taken up by the CCs for energy metabolism and oocyte energy supply (Sanchez-Lazo et al., 2014). Purportedly, this is closely bound up with mitochondria function and oxidative damage (Bradley and Swann, 2019). Raviv et al. (Raviv et al., 2020) revealed the correlation of increased lipid droplets in CCs and reduced pregnancy rates in *Homo sapiens*, hence presenting the importance of lipid metabolism in COCs. Currently, there have been growing attempts to increase the developmental potential of oocytes or COCs cultured *in vitro* by adding different kinds of fatty acids and metabolites (Boruszewska et al., 2015; Marei et al., 2017).

Ion transfer is one of the most fundamental functions of GJs in oogenesis (Tosti et al., 2013). The electrical cross-links between CCs and oocytes remain high at the end of follicular development, especially during meiosis (Carvacho et al., 2018). It is reasonable to speculate that a large amount of ion exchange is associated with intense metabolic activity (e.g., meiosis), and Na^+^, K^+^, and Ca^2+^ are the most common ions involved in membrane permeability and mitochondrial activity. The specific role of ions in folliculogenesis has not been fully elucidated.

Ovulation is a rather complex process that also includes morphologic changes in COCs (Robker et al., 2018). Besides rapidly producing extracellular matrix, CCs also adopt a transient increase in adhesive capacity during ovulation (Akison et al., 2012). AJs might also increase the adhesion of CCs. Komatsu and Masubuchi (Komatsu and Masubuchi, 2018) luckily observed the elongated projections of COCs pointing to a specific direction at ovulation. It’s reasonable to believe that CCs are moving in this direction, as well as pulling the oocyte out with them because of the physical connection between them.

### 3.4 Effects on cumulus cells: Proliferation and differentiation

Oocyte secreted factors (OSFs) are the main means of intercellular communication in the COC (Matzuk et al., 2002; Alam and Miyano, 2020). Oocytes can release OSFs by microvilli, thus shortening the time needed to diffuse into CCs and controlling the time and frequency of release. This is needed for the oocyte to achieve its central dominance in folliculogenesis (Zhang et al., 2021a). These factors, including BMP-15, GDF-9, and FGF-8 are involved in several important processes like regulation of germ cell migration to the gonadal crest and regulation of granulosa cell differentiation, proliferation, apoptosis, and luteinization. They also promote oocyte glycolysis required for oocyte metabolism and regulate the follicular growth rate (Sugiura et al., 2007; Chang et al., 2016).

OSFs secreted by oocytes stimulate granulosa cell proliferation, a process that requires the involvement of TGF-β, GDF9-I type I receptors (ALK4/5/6), and BMP-II receptors on the cell membrane and the activation of the SMAD2/3 or SMAD1/5/8 pathways (Dragovic et al., 2007; Hobeika et al., 2019; Hobeika et al., 2020). OSFs, especially GDF-9, have long been regarded as the main determinants of granulosa cell differentiation (Elvin et al., 1999; Su et al., 2004). Hsueh and his team clarified that GDF-9 could interact with BMP-II receptors and ALK5, and then activate the downstream SMAD2/3 pathways. They also revealed that GDF-9 could partially control the differentiation and proliferation of granulosa cells (Mazerbourg et al., 2004; Mazerbourg and Hsueh, 2006; Spicer et al., 2008). During the antral follicular phase, granulosa cells surrounding the oocyte differentiate into CCs under paracrine interactions with OSFs, which also maintain the phenotype of CCs and promote COC expansion (Gilchrist et al., 2004; Latham et al., 2004).

Based on the structure of microvilli, we can assume that OSFs are released under oocyte control and then they diffuse across the ZP to take effect. However, this theory leaves unanswered the question of why mural granulosa cells are not affected if OSFs can diffuse and even cross the ZP into the follicular fluid. Already differentiated MGCs could also form TZPs when cocultured with TZP-free denuded oocytes *in vitro* (Fushii et al., 2021). This suggests that there must be a mechanism that ensures the right differentiation of granulosa cells *in vivo*. A new perspective by Baena and Terasaki (Baena and Terasaki, 2019) proposes that OSFs do not take effect until the filopodia of granulosa cells contact the oocyte. A new look at the mechanism of granulosa cell differentiation is interesting. Using immunohistochemistry, Komatsu and Masubuchi (Komatsu and Masubuchi, 2018) found GDF-9 localized in the oocytes, cumulus cells, and COCs. They proposed that the distribution of OSFs was limited in COCs so that the differentiation of granulosa cells could be controlled. Does this finding challenge Baena and Terasaki’s theory? We think that in a complementary way the former explains the beginning of differentiation, whereas the latter reinforces the idea of a subsequent long-term communication process.

**FIGURE 3:**
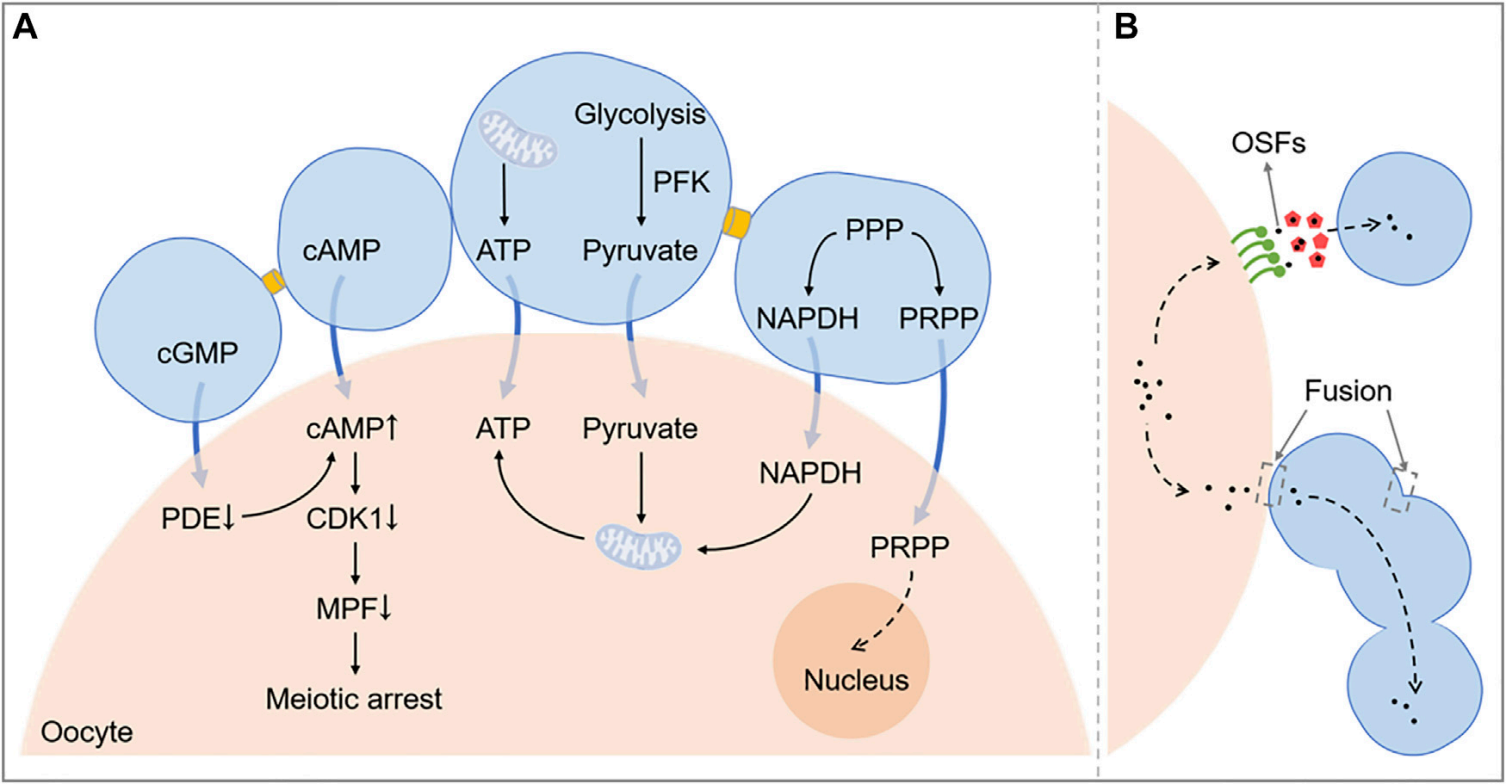
A schematic diagram exhibiting the function of intercellular communication in COC. Figure 3A shows related pathways of metabolism and meiosis between the oocyte and cumulus cells. Figure 3B shows that OSFs can be transported by EVs or directly get into cumulus cells through the membrane fusion. PPP, Pentose phosphate pathway; NADPH, Nicotinamide adenine dinucleotide phosphate; cAMP, Cyclic adenosine monophosphate; cGMP, Cyclic guanosine monophosphate; CDK1, Cyclin-dependent kinase 1; OSFs, oocyte secreted factors; PRPP, phosphoribosyl pyrophosphate; PDE, phosphodiesterase; MPF, maturation promoting factor; ATP, adenosine triphosphate.

It has been shown that OSFs can inhibit cell apoptosis by increasing the expression of Bcl-2 and inhibiting the expression of Bax and Caspase-3 in CCs (Moussa et al., 2018). It has also been suggested that oocytes can prevent follicular luteinization (el-Fouly et al., 1970). The removal of oocytes from COCs cultured *in vitro* leads to a significant increase in LHR and CYP11A1 induced by FSH and progesterone synthesis, which are the markers of luteinization. Further studies confirm that oocytes prevent CC luteinization by secreting OSFs to regulate steroid and inhibin synthesis and suppress LHR expression (Gilchrist et al., 2008; Gupta et al., 2017). An overview of these effects in COCs described above is shown in Figure 3.

## 4 Hormonal regulation of intercellular communication in COC

Méduri et al. (Méduri et al., 2002) observed FSH (but not LH) receptors in oocytes, but despite their observations, it is commonly believed that oocytes lack gonadotropin receptors (Yamoto et al., 1992; Minegishi et al., 1997a; Minegishi et al., 1997b; Lan et al., 2014). In the hypothalamic-pituitary-ovarian axis, oocytes lack responsiveness to gonadotrophins, while the granulosa cells are rich in FSH receptors and follicular theca cells are rich in LH receptors (Minegishi et al., 1997a; Minegishi et al., 1997b), so current studies have mostly focused on the intercellular communication in COC.

### 4.1 Regulation on gap junctions

The regulation of GJs by gonadotropins has been more thoroughly studied, and routinely there are hormones such as FSH and LH involved in it. FSH can upregulate CX43 expression through the PKA pathway, and it has recently been shown that FSH promotes CX43 assembly in the membrane through the WNT2/CTNNB1 pathway (Wang et al., 2013; Moussa et al., 2018). CTNNB1 is a protein in adherens junctions, thus corroborating the association between AJs and GJs. LH, on the other hand, inhibits CX43 expression (Kalma et al., 2004), but LH rises more slowly and steeply than FSH during normal follicle development, so GJs increase continuously before the follicle grow to a larger one. As mentioned previously, LH can also promote CX43 phosphorylation *via* the MAPK/PKA pathway, which causes changes in its spatial structure and a decrease in cAMP and cGMP transport to the oocyte; the addition of MAPK inhibitors in some studies significantly improved the LH-induced decline of CX43 expression (Kalma et al., 2004; Norris et al., 2008).

What’s more, androgen may also have an effect on GJs (Mohd Kamal et al., 2019). Clinical studies in PCOS patients have found a correlation between androgen and connexin expression, the expression of CX43 also increased in cultured granulosa cells by adding androstenedione, but the specific regulatory mechanisms have not been studied.

Recently, it was found that estradiol mediates the rapid downregulation of granulosa cell communications *via* the G protein-coupled receptor (GPR30) (Zhang et al., 2019). This process may involve the EGFR-ERK1/2 signalling pathway and be associated with phosphorylation of the CX43 protein.

### 4.2 Regulation on microvilli

Zachos et al. (Zachos et al., 2004; Zachos et al., 2008) found that estrogen could increase the number and height of microvilli on oocytes. Estrogen could also regulate the expression of alpha-actinin and the localization of ezrin-phosphate and SLC9A3R1 on the oocyte membrane, which is required for microvilli development. Bourdais et al. (Bourdais et al., 2021). Proposed that cofilin played a key role in oocyte actin network homeostasis and microvilli regulation. Another study revealed that Ano1, a Ca^2+^-activated Cl^−^ channel, could increase the length of microvilli *via* ERM-protein-dependent linkage to the cytoskeleton (Courjaret et al., 2016). In the study by Zhang et al. (Zhang et al., 2021a) the microvilli formation gene RADIXIN (RDX), an ERM family member, was found to be critical in microvilli formation. When RDX was silenced, the oocyte no longer formed microvilli, follicle development was retarded, and CC apoptosis increased. Interestingly, the TZPs in COCs were correspondingly reduced after RDX silencing, which might be partly due to the decreased number of CCs or interference of OSFs in TZP formation.

## 5 Applications of intercellular communications and future perspectives

Exploring cell interactions during follicular development can help us better understand the pathogenesis of related diseases such as polycystic ovary syndrome (PCOS) and primary ovarian insufficiency (POI). Zhao et al. (Zhao et al., 2010) found significantly reduced GDF-9 expression in CCs of patients with PCOS along with immature luteinization. Liu et al. (Liu et al., 2020) found a significant decreased expression of GJA1 mRNA (CX43 encoding gene) in COCs at the germinal vesicle stage of patients with PCOS, implying the impaired GJ function in PCOS. Decreased expression of GJs and connexins (including CX43 and CX37) and increased apoptosis of CCs were observed in the ovaries of type I diabetic mouse models (Ratchford et al., 2008). PCOS can be accompanied by several endocrine disorders including diabetes and obesity, perhaps suggesting the involvement of GJs in the abnormal follicular development seen in PCOS. Impaired expression of COC structural constituents might correlate to cumulus expansion and anovulation in PCOS patients, which is controlled by AREG and GDF-9 (Patil et al., 2022). Abbassi et al. (Abbassi et al., 2021) found that the epidermal growth factor receptor signaling pathway mediates the retraction of TZPs at ovulation, which might also contribute to the loss of communication in diseases like PCOS and POI. Bianchi et al. (Bianchi et al., 2015) suggested that reproductive aging and *in vitro* aging (due to extended culture) both could decrease the number of microvilli, leading to a poorer oocyte competence.

The researchers compared the EV contents in follicular fluid from different age groups and found some differences (da Silveira et al., 2012). The number and function of TZPs in COCs decreased in the elderly group (Zhang et al., 2022). The factors directly related to reproductive aging are the structure and function of mitochondria and the copy number of their DNA (Babayev et al., 2016). By studying changes in cell interactions, we could tell which synthesized substances are impaired in oocytes and CCs because of aging. A recent study constructed a ceRNA network with regard to exosomes in follicular fluid and revealed a variety of lncRNAs related to the pathogenesis of PCOS (Bai et al., 2022). In general, many respects of the PCOS pathogenesis, like lipid metabolism and chronic inflammation, were included and united into one integrity. Despite that PCOS is a systemic disease, it’s closely related to the homeostasis of intercellular environment in the single follicle, and EVs are the critical factor. The functions of different types of intercellular communications and their roles in reproductive diseases are briefly summarized in Table 1.

**TABLE 1:** Schematic description of the functions of intercellular communications and their roles in related reproductive diseases. TZP, transzonal projection; CC, cumulus cell; OSF, oocyte secreted factor; PCOS, polycystic ovary syndrome; POI, primary ovarian insufficiency.

Types	Functions	Role in reproductive disease
Transzonal Projections	Transportation of paracrine factors, signaling molecules and metabolites	Decreased numbers and impaired function in POI
Gap junctions	Channels at the tip of TZPs and between CCs	Decreased expression in PCOS
Microvilli	Contacting with TZPs; releasing OSFs	Decreased numbers in PCOS
Extracellular vesicles	Transportation of proteins, lipids and nucleic acids; maintaining follicular homeostasis	ceRNA network in PCOS related to lipid metabolism, chronic inflammationetc.
Cell membrane fusion	Sharing cytoplasm; ovulation	None so far

Based on the available studies, what we can affirm is the facilitative role of intercellular communications in folliculogenesis, and using this can also help us develop assisted reproduction techniques (ART). Fresh follicles were found to have higher levels of CX43 expression than vitrified follicles, and CX43 expression levels were also significantly lower in follicles cultured *in vitro* for 4 days (Bus et al., 2021), therefore, preservation and promotion of communications in cultured follicles may increase their quality in IVM. Kawai and Shimada found that during vitrification, the attachment of the oocyte to CCs was impaired because of the loss of cadherin adhesion molecules, which was critical for AJs (Kawai and Shimada, 2020). By treating ovaries briefly with collagenase before vitrification, they successfully preserved AJs and maintained the morphology of oocytes, suggesting a new potential approach for raising the functions and viability of cryopreserved ovaries. About 40% of oocytes after vitrification would have alteration of microvilli (Palmerini et al., 2014), indicating that the promotion or maintenance of microvilli during vitrification and warming may contribute to better fertilization, implantation and pregnancy rates.

EVs have been used to treat some female reproductive diseases, repair injured endometrium, regulate immunity and inflammation, and repress GC apoptosis (Sun et al., 2019; Ding et al., 2020; Li et al., 2020; Javadi et al., 2022). A randomized clinical trial showed that heterologous follicular fluid and supernatant of CCs could mimic the intact follicular microenvironment and improve the outcomes of immature oocytes in PCOS (Madkour et al., 2018). Addition of EVs could also aid in enhancing egg quality in IVM, although it has not yet been authoritatively proven.

Recently, single-cell sequencing studies on follicular development gene expression profiles have gradually emerged to reveal the molecular relationship between oocytes and granulosa cells during folliculogenesis and related diseases (Zhang et al., 2018; Fan et al., 2021; Long et al., 2022). These works provide a new perspective beyond the traditional direct observation of cell-to-cell interactions for future research.

## 6 Conclusion

In mammals, ovulation is a long and arduous task, requiring the fine cooperation of individual cells. Intercellular communications in the follicles are extensive and complex. TZPs and GJs are the most basic interactions that pave way for the survival and development of the oocyte. Microvilli, extracellular vesicles, cell membrane fusion, and other uncovered interactions broaden the regulation between oocyte and cumulus cells, to make the COC a true entirety. The importance of intercellular communications is self-evident, but the detailed mechanisms have long been perplexing.

In conclusion, this review details several types of intercellular communications in COCs, both already identified and newly discovered ones, as well as their functions and regulations. These complex mechanisms are the basis for our exploration of human physiology and help us to move further down the path of disease treatment and assisted reproduction.

## Glossary

CC: Cumulus cell
MGC: Mural granulosa cell
FSH: Follicle-stimulating hormone
FSHR: FSH receptor
LH: Luteinizing hormone
LHR: LH receptor
ZP: Zona pellucida
COC: Cumulus-oocyte complex
TZP: Transzonal projection
GJ: Gap junction
CX: Connexin
AJ: Adherens junction
TJ: Tight junction
ZO: Zonula occludens
EV: Extracellular vesicle
PPP: Pentose phosphate pathway
PFK: Phosphofructokinase
NADPH: Nicotinamide adenine dinucleotide phosphate
OSF: Oocyte secreted factor
cAMP: Cyclic adenosine monophosphate
cGMP: Cyclic guanosine monophosphate
CDK1: Cyclin-dependent kinase 1
PKA: protein kinase A
NPPC: Natriuretic peptide type C
NPR2: Natriuretic peptide receptor 2
BMP: Bone morphogenetic protein
GDF: Growth differentiation factor
FGF: Fibroblast growth factor
TGF: Transforming growth factor
ALK: Anaplastic lymphoma kinase
SMAD: Small mothers against decapentaplegic
PCOS: Polycystic ovary syndrome
POI: primary ovarian insufficiency
GPR30: G protein-coupled receptor 30
RDX: RADIXIN
AREG: Amphiregulin
ART: Assisted reproduction technique
IVM: *In vitro* maturation.
